# Mussel-Inspired Fabrication of SERS Swabs for Highly Sensitive and Conformal Rapid Detection of Thiram Bactericides

**DOI:** 10.3390/nano9091331

**Published:** 2019-09-17

**Authors:** Jun Liu, Tiantian Si, Lingzi Zhang, Zhiliang Zhang

**Affiliations:** 1 State Key Laboratory of Biobased Material and Green Papermaking, Qilu University of Technology (Shandong Academy of Sciences), Jinan 250353, China; liujun6621@126.com; 2 School of Light Industry Science and Engineering, Qilu University of Technology (Shandong Academy of Sciences), Jinan 250353, China; 3 Key Laboratory of Fine Chemicals in Universities of Shandong, School of Chemistry and Pharmaceutical Engineering, Qilu University of Technology (Shandong Academy of Sciences), Jinan 250353, China; sttsitiantian@163.com (T.S.); 1139195967@qq.com (L.Z.)

**Keywords:** polydopamine, in-situ grown, flexible SERS swabs, on-site detection, thiram bactericides

## Abstract

As an important sort of dithiocarbamate bactericide, thiram has been widely used for fruits, vegetables and mature crops to control various fungal diseases; however, the thiram residues in the environment pose a serious threat to human health. In this work, silver nanoparticles (AgNPs) were grown in-situ on cotton swab (CS) surfaces, based on the mussel-inspired polydopamine (PDA) molecule and designed as highly sensitive surface-enhanced Raman scattering (SERS) swabs for the conformal rapid detection of bactericide residues. With this strategy, the obtained CS@PDA@AgNPs swabs demonstrated highly sensitive and reproducible Raman signals toward Nile blue A (NBA) probe molecules, and the detection limit was as low as 1.0 × 10^−10^ M. More critically, these CS@PDA@AgNPs swabs could be served as flexible SERS substrates for the conformal rapid detection of thiram bactericides from various fruit surfaces through a simple swabbing approach. The results showed that the detection limit of thiram residues from pear, grape and peach surfaces was approximately down to the level of 0.12 ng/cm^2^, 0.24 ng/cm^2^ and 0.15 ng/cm^2^ respectively, demonstrating a high sensitivity and excellent reliability toward dithiocarbamate bactericides. Not only could these SERS swabs significantly promote the collection efficiency of thiram residues from irregular shaped matrices, but they could also greatly enhance the analytical sensitivity and reliability, and would have great potential for the on-site detection of residual bactericides in the environment and in bioscience fields.

## 1. Introduction

Thiram, an important member of dithiocarbamate bactericides, has been widely used in agriculture fields to protect fruit, vegetable, ornamental plants and crops from various fungal diseases [1,2,3]. In addition, it is also used as a protective fungicide and animal repellent to preserve mature fruits and vegetables during storage and shipment [4]. The biological activity of thiram is principally dependent on the chemical reactivity of dithiocarbamate groups in the molecules, which could react with the HS-containing enzymes and coenzymes of fungal cells, and thus effectively block their catalytic activity [5]. However, the biological toxicity could cause serious skin, eye and mucosa illness induced by exposure to thiram; additionally, an uptake of thiram from fruits and vegetables could induce dyspnea, ataxia, convulsions, and severe fetal malformations [6]. Otherwise, thiram is suspected to be a potential carcinogen and teratogen to the human body upon chronic exposure. More seriously, it is very difficult to remove thiram from the natural environment due to its poor solubility, and it could give rise to soil pollution and even seep into groundwater [4,7,8].

Therefore, the problems of thiram residues have attracted extensive public attention, and the U.S. Environmental Protection Agency (EPA) have set up a series of laws and regulations to restrict the level of thiram bactericides released into the environment [9]. To date, a variety of laboratory analytical methods, such as spectrophotometry, voltammetry, chromatography, capillary electrophoresis, enzyme-linked immunosorbent assay (ELISA) and enzyme inhibition have been exploited to detect the residues of thiram bactericides in the environment [10,11]. Many of these methods are highly sensitive and accurate in detecting trace thiram bactericides; however, the sample extraction processes are time-consuming and require the extensive manual handling of toxic samples as well as large amounts of organic solvent [11]. In addition, the instruments employed in the above methods are generally complicated and high-cost, and could not be used for the on-site monitoring of thiram residues [4,12].

Owing to its high sensitivity, excellent selectivity, reproducible ability and nondestructive data acquisition [13,14,15], SERS provides a robust and powerful detection technique for efficaciously identifying chemical and biological species in the fields of environmental monitoring [16], explosives detection [17,18] and biomedical science [19]. Thus, compared to the above mentioned analytical methods, SERS would be an excellent technique for the label-free and sensitive detection of thiram, and it would be expected to be one of the best candidates for on-site bactericide detection in real applications [12,20]. Despite these exciting advantages, most current SERS substrates are based on various metal nanostructures immobilized on a series of rigid supporting substrates, such as glass sheets, silicon wafers and porous alumina [21]. These rigid SERS substrates severely lack in flexibility and prevent a conformal contact with topologically complex surfaces, which results in a low collection efficiency and which drastically restricts their in-situ analyte detection from the irregular surface [22].

Recently, the emerging flexible SERS substrates have provided an excellent opportunity to achieve the sensitive SERS detection of trace target molecules from uneven or rugged surfaces [22,23,24]. For example, various metallic nanostructures have been intensively utilized and have decorated the papers, cottons, sponges and copolymers to serve as SERS flexible substrates [17,25,26]. Due to the high specific surface area, multitudinous porosity and good mechanical strength, these SERS substrates demonstrate a good flexibility and efficiency for target molecule detection via the swabbing method. Although the flexible SERS substrates demonstrate a fairly enormous application prospect, it still remains a big challenge to collect the targets from a real-world surface and achieve a highly-sensitive SERS detection [18,23]. Consequently, it is very urgent to exploit an approach to fabricate flexible and soft SERS substrates for the on-site analysis of trace bactericide residues [4,12].

As an important sort of biomimetic macromolecule, polydopamine (PDA) has abundant amine and catechol groups on the molecular surface, and it demonstrates similar molecule structures and properties to marine mussels, such as a superior self-polymerization, in-situ reduction and special recognition capability [27,28]. In particular, PDA molecules could readily form a functional layer on the various substance surfaces through self-polymerization, which provides an efficient and simple method for surface modification [28,29,30]. More importantly, the catechol and amine groups on PDA molecule surfaces have a strong complexation with various metallic cations and spontaneously in-situ reduce them into metallic nanostructures by oxidizing catechols into the corresponding quinine groups [31,32,33]. Consequently, PDA could serve as a versatile molecule platform for synthesizing various metal nanostructures on the desirable scaffold, and for providing a promising opportunity to fabricate flexible SERS substrates for the on-site analysis of thiram bactericide residues from the real-world surface.

Herein, we present a convenient and rational strategy for the in-situ growth of abundant AgNPs onto cotton swab surfaces based on the mussel-inspired surface chemistry, and we explore them as flexible SERS swabs for the detection of trace thiram residues. With this design, abundant AgNPs as SERS hot spots were grown in-situ onto the surface of cotton swabs by controlling the redox reaction between catechol groups and [Ag(NH_3_)^2^]^+^ cations in the solution. Due to the significant electromagnetic coupling effect generated by the densely-packed AgNPs, these SERS swabs demonstrated a high sensitivity and excellent reliability, and the SERS signal of the NBA molecule could be detected at a concentration level as low as 1.0 × 10^−10^ M. Moreover, the fabricated SERS swabs could be directly employed as flexible SERS substrates for the conformal detection of thiram bactericides from various fruit surfaces through a simple swabbing approach. It was verified that the detection limit of thiram residues from pears, grapes and peaches was approximately down to 0.12 ng/cm^2^, 0.24 ng/cm^2^ and 0.15 ng/cm^2^, respectively. These SERS swabs could not only sharply increase the analyte collection efficiency, but also be a simple and efficient platform for the on-site detection of trace thiram bactericide residues. The scalable and reproducible approach would have great potential for environmental monitoring and the ultrasensitive detection of trace harmful chemicals in the agriculture, environment and bioscience fields.

## 2. Materials and Methods

### 2.1. Chemicals and Materials

Silver nitrate was purchased from the China National Pharmaceutical Group Corporation (Shanghai, China). Dopamine (DA), tris-base, Nile blue A (NBA) and thiram were obtained from Sigma-Aldrich (St. Louis, MO, USA). Cotton swabs were purchased from the local supermarket (Jinan, China). Ethanol and acetone were from Beijing Chemical Co. Ltd. (Beijing, China), and the other chemicals were analytical or high-reagent grade. The ultrapure water (18.2 MΩ) produced by a Milli-Q system was used throughout the experiments.

### 2.2. Decoration Cotton Swabs with Dopamine

The cotton swabs were ultrasonically cleaned by ethanol, acetone and deionized (DI) water, respectively, and dried at 60 °C in an oven. Subsequently, the cleaned cotton swabs were immersed in Tris-HCl buffer solution with DA molecules and reacted at pH = 8.5 for a certain time. Owing to a spontaneous oxidative polymerization, a functional PDA layer was formed on the surface of the cotton swabs. Finally, the excess DA molecules on the cotton swabs’ surfaces were removed by washing adequately with ethanol and DI water, and dried under nitrogen atmosphere for further analysis. The obtained composites were denoted as the CS@PDA swabs.

### 2.3. In-Situ Grown AgNPs on the CS@PDA Swabs Surface

In order to realize in-situ grown AgNPs on the CS@PDA surface, the obtained CS@PDA swabs were further immersed into the fresh [Ag(NH_3_)_2_]^+^ solution with continuous stirring. Due to the complexation and in-situ reduction capacity, the catechol and amine groups on the PDA surface could coordinates with the [Ag(NH_3_)_2_]^+^ cations and spontaneously in-situ reduce them into the respective AgNPs. By controlling the reaction time, a controlled density of AgNPs was generated on the surface of the CS@PDA swabs. The obtained composites were washed adequately with DI water to remove the redundant [Ag(NH_3_)_2_]^+^ cations, and denoted as the CS@PDA@AgNPs swabs.

### 2.4. Sensitivity and Reliability of CS@PDA@AgNPs Swabs with NBA as Probe Molecules

To verify the performance of CS@PDA@AgNPs swabs as flexible SERS substrates, NBA was used as probe molecules to evaluate the sensitivity and reproducibility. The obtained CS@PDA@AgNPs swabs were immersed into NBA solution with the range concentration of 10^−3^ M∼10^−11^ M. After that, the CS@PDA@AgNPs swabs were dried at room temperature for the SERS analysis.

### 2.5. SERS Detection of Thiram Residues with CS@PDA@AgNPs Swabs

To investigate the limit of bactericide detection, thiram powder was dissolved in ethanol to form a standard stock solution and sequentially diluted with DI water into a series of thiram solutions in the range of 10^−3^ M–10^−^^7^ M concentrations. All fruits used in the SERS analysis, such as pears, grapes and peaches, were adequately washed with ultrapure water. Subsequently, the thiram solutions with the concentration of 10^−3^ M–10^−7^ M were sprayed onto the surface of the above abluent pears, grapes and peaches, respectively, and the spray area was controlled in 1 cm × 1 cm square centimeters. Then, the thiram bactericides on the surface of the pears, grapes and peaches were carefully collected by swabbing with the flexible CS@PDA@AgNPs swabs. As the solvent absolutely evaporated, the CS@PDA@AgNPs swabs were subjected to SERS detection.

### 2.6. Characterization

The morphology and energy-dispersive spectroscopy (EDS) of the CS, CS@PDA and CS@PDA@AgNPs swabs were conducted on a Hitachi S-8220 scanning electron microscope (Hitachi, Japan). The X-ray diffraction (XRD) patterns of the above samples were performed on a D8 Advance X-ray diffractometer (Bruker, Germany). The X-ray photoelectron spectroscopy (XPS) was collected on Thermo Scientific ESCALab Xi^+^ with 200 W monochromatic Al Kα radiation (Thermo Fisher, USA). All Raman measurements were conducted on a Renishaw inVia9 Raman Microscope (Renishaw, UK) and 50× objective, and 532 nm laser irradiation was applied to focus onto a spot in the samples with approximately 1 μm diameter. The intensity of the irradiation laser was controlled at 1 mW to activate the samples during the whole process, and all SERS signals were gathered at about 1 s. For every swab, three different points were taken to conduct the respective thiram SERS measurement.

## 3. Results and Discussion

**Figure 1:**
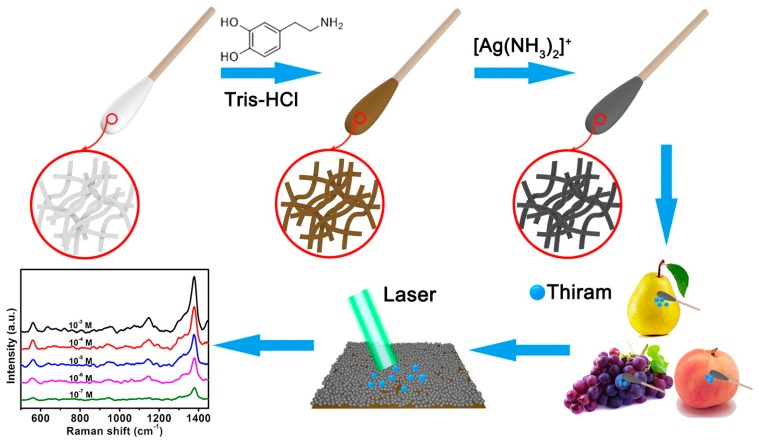
The schematic of the fabrication of CS@PDA@AgNPs swabs for the conformal rapid SERS detection of thiram bactericide residues.

As a three-dimensional flexible material, cotton swabs have large specific surfaces and a superior permeability, and these characters enable them to be an appropriate candidate for the fabrication of flexible SERS substrates [34]. Simultaneously, the excellent flexibility enables them to have a good conformal contact with the irregular surface and to achieve the detection of surface contaminants from various complex surfaces [22,35]. Figure 1 illustrates our design to fabricate the flexible SERS substrates for the conformal rapid detection of thiram bactericide residues through in-situ grown AgNPs onto the cotton swabs, based on the mussel-inspired surface chemistry. With this strategy, the precleaned cotton swabs were firstly immersed into DA solution, and adhesive and versatile PDA layers were spontaneously formed on the cotton swabs’ surfaces by DA self-polymerization. Owing to the strong complexation capacity, the amino and catechol groups on the PDA layers could be complexed with [Ag(NH_3_)_2_]^+^ cations after being dipping into the silver ammonia solution. Subsequently, abundant AgNPs as SERS hot spots were in-situ grown onto the surface of the cotton swabs via a silver cations reduction by oxidizing catechol into the corresponding quinine groups. The obtained CS@PDA@AgNPs swabs could be directly utilized as SERS swabs to collect the thiram bactericides from the surface of pears, grapes and peaches by a simple swabbing approach. Due to the significant electromagnetic coupling effect and superior collection efficiency, these flexible SERS swabs could achieve a high SERS detection sensitivity and reliability for trace thiram bactericides from various complex surfaces.

**Figure 2:**
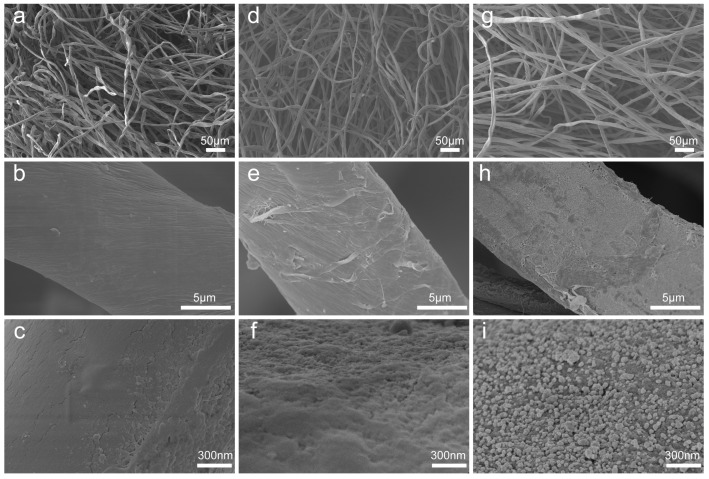
SEM images of the (**a**–**c**) original cotton swabs, (**d**–**f**) CS@PDA swabs and (**g**–**i**) CS@PDA@AgNPs swabs, respectively.

The morphology and nanogaps of the CS@PDA@AgNPs swabs were closely related to the SERS performance and determined the sensitivity and reliability of the thiram bactericides from a real-world surface [36]. To exhibit the respective variation of the surface morphologies and elements, the CS, CS@PDA and CS@PDA@AgNPs swabs were investigated by SEM and EDS. As shown in Figure 2a–c, the fibers of the cotton swabs presented three-dimensional net structures, and these characteristics enabled the cotton swabs to possess a superior permeability. In addition, the surface of the original fibers in the cotton swabs was very smooth and exhibited no obvious nanostructures. After being immersed in the DA solution, adhesive and versatile PDA layers were spontaneously formed on the cotton swab surfaces via DA self-polymerization (Figure 2d–f), and the CS@PDA swabs became rough, brown and dark (Figure S1a). As they further reacted with the silver ammonia solution, a large number of nanostructures and nanogaps as SERS hot spots were in-situ formed and tightly covered on the surface of the cotton swabs (Figure 2g–i). Due to the ultrahigh adhesion properties of PDA functional layers [37], the AgNPs demonstrated an excellent interfacial interaction with the swabs’ fiber surfaces, which suggested that the CS@PDA@AgNPs swab could serve as a robust SERS substrate for highly sensitive detection.

Furthermore, the original CS, CS@PDA and CS@PDA@AgNPs swabs were also characterized by EDS, and the respective results were shown in Figure S1b–d. When comparing the EDS peaks of the CS@PDA@AgNPs swabs with the original CS and CS@PDA swabs, an obvious peak of silver element emerged and demonstrated a significant intensity, which suggested that a mass of AgNPs were formed on the surface of the CS@PDA@AgNPs swabs. At the same time, four characteristic diffraction peaks at 2θ = 37.86°, 44.12°, 64.28°and 77.20° clearly emerged in the XRD spectrum of the CS@PDA@AgNPs swabs (Figure S2), corresponding to the (110), (200), (220) and (311) planes of the cubic silver crystal (JCPDS No.83-0718) [27,38]. From all of the above analyses, a controllable density of AgNPs was successfully in-situ grown on the surface of the CS@PDA@AgNPs swabs, based on mussel-inspired PDA surface chemistry.

**Figure 3:**
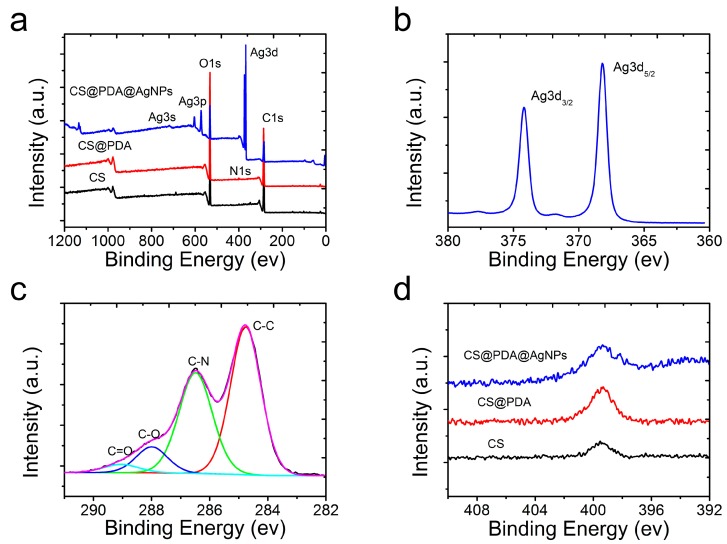
(**a**) XPS survey of the original cotton swabs, CS@PDA swabs and CS@PDA@AgNPs swabs. (**b**) High-resolution Ag3d spectrum in the CS@PDA@AgNPs swabs. (**c**) Narrow-scan XPS spectra of C1s in the CS@PDA@AgNPs swabs. (**d**) The changes of the N1s spectrum in the original cotton swabs, CS@PDA swabs and CS@PDA@AgNPs swabs.

In order to further prove the changes of the surface component after the mussel-inspired PDA biomimetic modification, the original CS, CS@PDA and CS@PDA@AgNPs swabs were characterized by an X-ray photoelectron spectrometer. As shown in Figure 3a, compared with the XPS survey spectra of the original CS and CS@PDA swabs, the characteristic emission peaks of silver element, such as Ag3s (719 eV), Ag3p (573 eV and 604 eV) and Ag3d (368 eV and 374 eV), obviously appeared in the XPS survey of the CS@PDA@AgNPs swabs, which proved that abundant AgNPs were formed on the CS@PDA surface through the in-situ reducing role of catechol groups. In addition, from the high-resolution Ag3d spectrum (Figure 3b), the binding energies for Ag 3d_5/2_ and Ag 3d_3/2_ were verified at 368.2 and 374.2 eV, respectively, and the approximate 6 eV splitting of the 3d doublet owing to the spin-orbit coupling further identified a single zero valence silver-element existence on the surface of the CS@PDA@AgNPs swabs [33]. Furthermore, the high-resolution C1s spectrum of the CS@PDA@AgNPs swabs was curved by four distinct peaks displayed at 289.05 eV, 288.15 eV, 286.5 eV and 284.8 eV (Figure 3c), which were attributed to the existence of carbonyl groups (C=O), the C–O groups, the C–N groups and saturated C–C bonding, respectively [27]. From Figure 3d, compared to the original cotton swab, the nitrogen peaks increased on the CS@PDA surface due to a functional PDA layer formation via DA self-polymerization. As the AgNPs were in-situ grown on the surface of CS@PDA and from the respective CS@PDA@AgNPs swabs, the intensity of nitrogen peaks demonstrated an obvious decrease. From the above analysis, all these XPS results were greatly consistent with the SEM and EDS results, indicating that flexible SERS swabs were successfully fabricated based on mussel-inspired surface chemistry.

**Figure 4:**
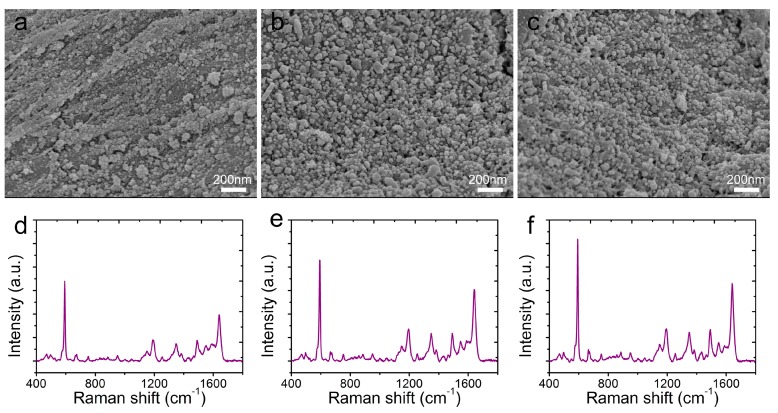
SEM images of the CS@PDA@AgNPs swabs after reacting with [Ag(NH_3_)_2_]+ anions for (**a**) 4 h, (**b**) 8 h and (**c**) 12 h. (**d**–**f**) The respective SERS spectra of NBA collected on the 4 h, 8 h and 12 h CS@PDA@AgNPs swabs.

The AgNPs morphology on the CS@PDA@AgNPs swabs was closely related to the reaction time, and determined the detection sensitivity and reliability [39]. In order to obtain the strongest SERS signals, the nanostructures and nanogaps from the CS@PDA@AgNPs swabs were controlled by the reaction time to regulate the AgNPs growth on the CS@PDA surface based on the mussel-inspired chemistry. From Figure 4a–c, initially, the AgNPs amount on the CS@PDA@AgNPs swab surfaces was small and randomly distributed due to a short reaction time (Figure 4a: 4 h). With an increase of the reaction time, the AgNPs density became enhanced as an increased number of [Ag(NH_3_)_2_]^+^ cations were in-situ reduced into AgNPs by the catechol groups (Figure 4b: 8 h). As the reaction time further increased to 12 h, abundant interstices and nanogaps were formed among AgNPs on the surface of the CS@PDA@AgNPs swabs (Figure 4c: 12 h). The corresponding SERS spectra of NBA as probe molecules (1 × 10^−3^ M) were collected from the above CS@PDA@AgNPs swabs. From Figure 4d–f, the SERS intensity obviously increased with the extension of the reaction time in the [Ag(NH_3_)_2_]^+^ solution, and it achieved a maximum SERS intensity when the reaction time was 12 h, which was due to the fact that a large quantity of SERS “hot spots” was generated on the CS@PDA@AgNPs swabs and gave a rise to strong electromagnetic enhancement [40]. As a result, in the following SERS experiments, the CS@PDA@AgNPs swabs fabricated at 12 h would be used as flexible SERS substrates for various detections.

**Figure 5:**
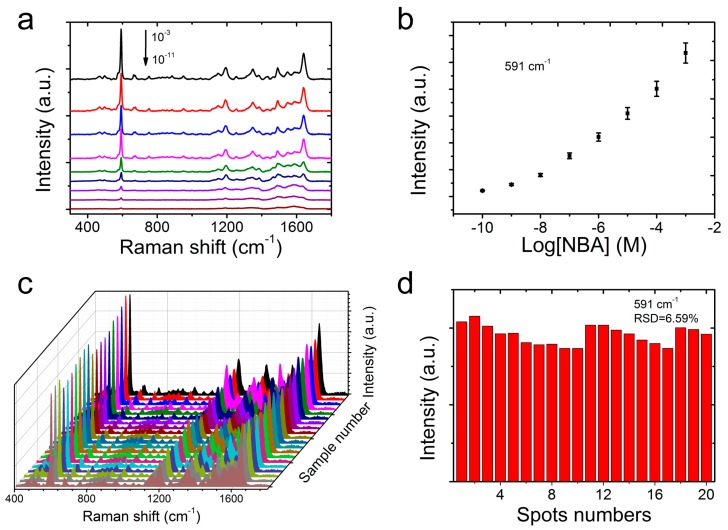
(**a**) The Raman spectra of the NBA probe molecules at different concentrations on the CS@PDA@AgNPs swabs. (**b**) The relationship between the signal intensity at 591 cm^−1^ of the NBA molecules and the corresponding logarithmic concentration. (**c**) The SERS homogeneity of the CS@PDA@AgNPs swabs from 20 randomly selected points. (**d**) The Raman intensity distribution of the NBA molecules at 591 cm^−1^ collected from 20 randomly selected points.

To further evaluate the SERS sensitivity, the CS@PDA@AgNPs swabs were immersed into an NBA solution with different concentration ranging from 1 × 10^−3^ to 1 × 10^−11^ M. After the CS@PDA@AgNPs swabs were dried, the responding SERS spectra were collected. From Figure 5a, the Raman signal intensity of the NBA molecules decreased promptly with concentrations ranging from 1 × 10^−3^ to 1 × 10^−11^ M. When the NBA concentration was lower than 1 × 10^−10^ M, the characteristic peaks, such as at 591 cm^−1^ and 1640 cm^−1^, could still be observed [41,42]. If the concentration further decreased to 1 × 10^−11^ M, the above characteristic Raman peaks of the NBA molecules could not be distinctly detected, and the limit of detection (LOD) for the NBA molecules was approximately 1 × 10^−10^ M. Furthermore, Figure 5b shows the relationship of the signal intensity of the NBA molecules with the solution concentration from 1 × 10^−3^ to 1 × 10^−10^ M. According to the characteristic peak intensities at 591 cm^−1^, the signal intensity of the NBA molecules was sharply enhanced with an increase of the corresponding logarithmic concentration, which was fairly favorable to the quantitative determination for target molecules.

In addition to a superior sensitivity, the homogeneity of the Raman signals was another crucial factor for the CS@PDA@AgNPs swabs as flexible substrates for achieving the credible results. In order to verify the homogeneity, more than 20 spots were randomly selected on the surface of the CS@PDA@AgNPs swabs, and the corresponding NBA SERS spectra were collected. Figure 5c shows that all of the selected spots showed almost identical Raman signals, and the relative standard deviation (RSD) of the SERS peak at 591 cm^−1^ was 6.59% (Figure 5d). On the basis of these statistical results, it was fully proven that the CS@PDA@AgNPs swabs fabricated with our design possessed a high sensitivity and homogeneity, and could be utilized as flexible SERS substrates for trace molecule detection.

**Figure 6:**
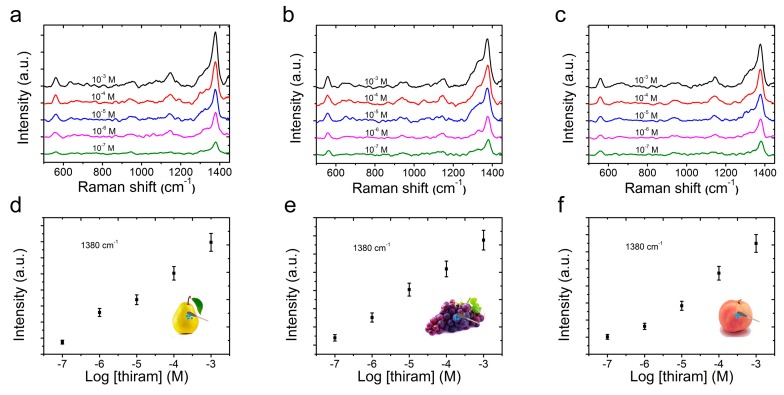
The SERS spectra of thiram with different concentrations collected by CS@PDA@AgNPs swabs through swabbing extraction on (**a**) pear, (**b**) grape and (**c**) peach surfaces. The relationship between the SERS peak intensity at 1380 cm^−^^1^ and the corresponding logarithmic concentration of thiram molecules on (**d**) pear, (**e**) grape and (**f**) peach surfaces through a swabbing extraction.

Due to the efficiency, sensitivity and practicality, swabbing was considered to be one of the most versatile sampling methods for a target molecule analysis from a real-world surface, and could greatly enhance the efficiency of a sample collection [34,43]. In order to elevate the applicability of CS@PDA@AgNPs swabs as flexible SERS substrates to collect thiram residues from multifarious fruits, thiram solutions with different concentrations ranging from 1 × 10^−3^ to 1 × 10^−7^ M were sequentially sprayed on the precleaned surfaces of pears, grapes and peaches. As the solutions were dried, the thiram residues were collected by the CS@PDA@AgNPs swabs via a surface swabbing method, and detected with the Renishaw inVia9 Raman Microscope. As shown in Figure 6a–c, the characteristic peaks of the thiram molecules, such as at 561 cm ^−1^ attributed to υ(S-S), 1147 cm^−1^ corresponding to ρ(-CH3) and υ(C-N), and 1380 cm^−1^ ascribed to υ(C-N), were clearly detected, which was consistent with the previous reports for the thiram SERS analysis [41,44]. At the same time, even if the thiram concentration decreased to 1 × 10^−7^ M, the above characteristic SERS peaks could still be clearly discerned, demonstrating a high SERS sensitivity for the CS@PDA@AgNPs swabs to detect thiram residues from various surfaces. Moreover, the Raman signals of thiram molecules at 1380 cm^−1^ were collected from a normal silicon wafer and the CS@PDA@AgNPs swabs, respectively, and were used to calculate the enhancement factor (EF). According to the previous calculation method [45], the thiram EFs on the pear, grape and peach surfaces could reach high values of up to 1.84 × 10^5^, 1.61 × 10^5^ and 1.75 × 10^5^, respectively, and these high EFs were fairly favorable for the CS@PDA@AgNPs swabs to achieve trace thiram residue detection.

Furthermore, Figure 6d–f demonstrates the relationship between the SERS peak intensity at 1380 cm^−1^ and the corresponding logarithmic concentration for the thiram bactericide from the surface of pear, grapes and peaches, respectively. According to the characteristic peak intensities at 1380 cm^−1^, the SERS intensity of thiram bactericide was rapidly enhanced with an increasing concentration, which contributed to an accurate detection of these bactericide molecules. A calculation using the concentration (1 × 10^−^^7^ M) and volume (5 μL) of thiram bactericide sprayed onto the pear surfaces showed that about 0.12 ng of the thiram sample was deposited on the pears’ surfaces on an area of 1 × 1 cm^2^, and that it was still effectively detected by this direct swabbing method. According to the above quantitative approach, thiram bactericides on the grapes’ and peaches’ surfaces were also tested, and these analytes could even be detected at an amount of 0.24 ng/cm^2^ and 0.15 ng/cm^2^. From the above analysis results, the fabricated CS@PDA@AgNPs swabs could be utilized as SERS swabs to collect thiram bactericides via a direct swabbing method, providing a satisfactory strategy for the quantitative analysis of various surface contaminants from a real-world surface [46].

**Figure 7:**
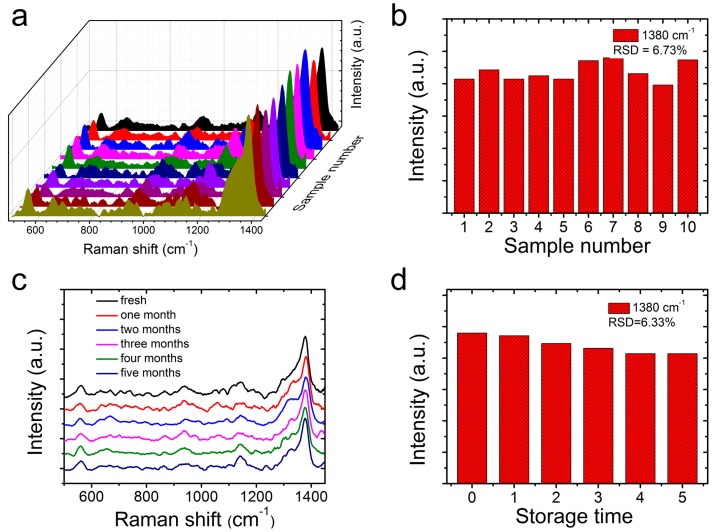
(**a**) The SERS homogeneity of thiram on the CS@PDA@AgNPs swabs from 10 random spots. (**b**) The corresponding intensity variation at 1380 cm^−1^ in the histogram. (**c**) The Raman spectrum of thiram from the CS@PDA@AgNPs swabs stored for 0–5 months. (**d**) The corresponding intensity variation at 1380 cm^−1^ in the histogram.

In addition to a high sensitivity, the SERS homogeneity of the CS@PDA@AgNPs swabs was another pivotal parameter for the achievement of a reliable SERS detection. In this work, the SERS homogeneity of the CS@PDA@AgNPs swabs to thiram bactericides was also investigated in detail. After carefully swabbing the thiram bactericide from the pear surface, we randomly selected 10 spots from the CS@PDA@AgNPs swabs and collected the respective SERS spectra. From Figure 7a, the selected 10 spots demonstrated almost uniform Raman spectra of thiram bactericide, and the RSD values of the Raman peaks at 1380 cm^−1^ were approximately 6.73% (Figure 7b), indicating a good SERS homogeneity. Moreover, as shown in Figure 7c,d, the intensity of the characteristic bands exhibited no obvious changes after storage for five months, and the RSD values of the Raman peaks at 1380 cm^−1^ were approximately 6.33%, which suggested that the CS@PDA@AgNPs swabs have an excellent SERS stability. From all of the above results, the fabricated CS@PDA@ AgNPs swabs possessed a high sensitivity and excellent homogeneity, and could serve as superior flexible SERS swabs to detect thiram bactericide residues via a direct swabbing method; furthermore, they would have great potential for the on-site detection of residual bactericides in the environment and in bioscience fields.

## 4. Conclusions

In conclusion, highly sensitive and homogeneous flexible CS@PDA@AgNPs swabs were fabricated through the in-situ growth of abundant AgNPs onto cotton swab surfaces, based on the mussel-inspired surface chemistry. Due to the strong electromagnetic coupling effect generated by the densely-packed AgNPs, the fabricated CS@PDA@AgNPs swabs demonstrated a high sensitivity and excellent reliability to NBA probe molecules, and the LOD was lower than a concentration level of 1.0 × 10^−10^ M. More crucially, the fabricated flexible CS@PDA@AgNPs swabs could achieve a rapidly conformal detection of thiram bactericides from various fruit surfaces via a direct swabbing approach, and demonstrated a high sensitivity and uniformity. Furthermore, the CS@PDA@AgNPs swabs provided a good relationship between the normalized Raman intensity and logarithmic concentration, and the LOD of the thiram residues from pears, grapes and peaches was approximately down to 0.12 ng/cm^2^, 0.24 ng/cm^2^ and 0.15 ng/cm^2^ respectively. These CS@PDA@AgNPs swabs would be excellent SERS swabs for the collection of various contaminants from a real-world surface and have a great potential for the detection of bactericide residues in the environment and in bioscience fields.

## Supplementary Materials

The following are available online at https://www.mdpi.com/2079-4991/9/9/1331/s1, Figure S1: (a) Optical images of the original cotton swabs, CS@PDA swabs and CS@PDA@AgNPs swabs. The responding EDS spectra of (b) the original cotton swabs, (c) the CS@PDA swabs and (d) the CS@PDA@AgNPs swabs respectively. Figure S2: XRD of the original cotton swabs, CS@PDA swabs and CS@PDA@AgNPs swabs. Figure S3: SERS spectra (black) of thiram on the CS@PDA@AgNPs swabs collected from (a) pears, (b) grapes, and (c) peaches surface, and the Raman spectra (red) of thiram on silicon wafer substrate.
