# Supplementary material for: Mussel-Inspired Fabrication of SERS Swabs for Highly Sensitive and Conformal Rapid Detection of Thiram Bactericides
Source: Nanomaterials (Basel). 2019 Sep 17;9(9):1331. doi: 10.3390/nano9091331 (PMC6781073; doi:10.3390/nano9091331)
Supplement: Supplementary file 1 [file nanomaterials-09-01331-s001.pdf]

Supplementary Materials

Mussel-Inspired Fabrication of SERS Swabs for Highly Sensitive and Conformal Rapid Detection of Thiram Bactericides

Jun Liu [1,2], Tiantian Si [3], Lingzi Zhang [3] and Zhiliang Zhang [1,3,*]

1 State Key Laboratory of Biobased Material and Green Papermaking, Qilu University of Technology (Shandong Academy of Sciences), Jinan, 250353, China

2 School of Light Industry Science and Engineering, Qilu University of Technology (Shandong Academy of Sciences), Jinan, 250353, China

3 Key Laboratory of Fine Chemicals in Universities of Shandong, School of Chemistry and Pharmaceutical Engineering, Qilu University of Technology (Shandong Academy of Sciences), Jinan, 250353, China

* Correspondence: zhzhl@iccas.ac.cn; Tel.: +86-053189631632

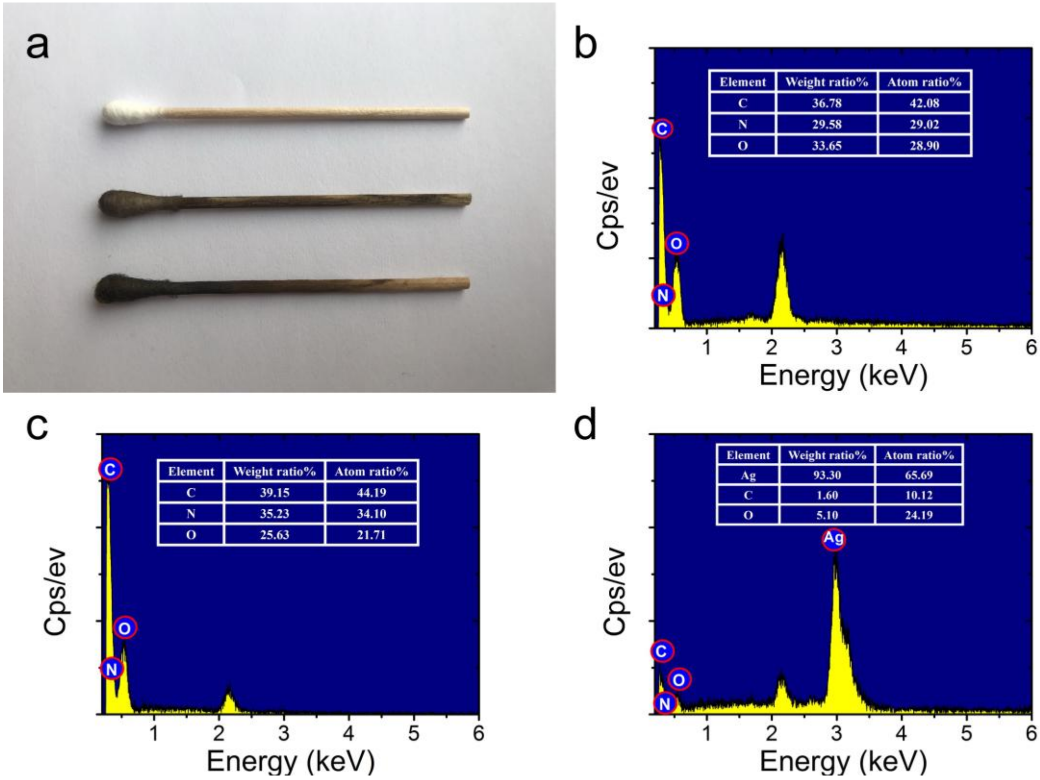

Figure S1. (**a**) Optical images of the original cotton swabs, CS@PDA swabs and CS@PDA@AgNPs swabs. The responding EDS spectra of (**b**) the original cotton swabs, (**c**) the CS@PDA swabs and (**d**) the CS@PDA@AgNPs swabs respectively.

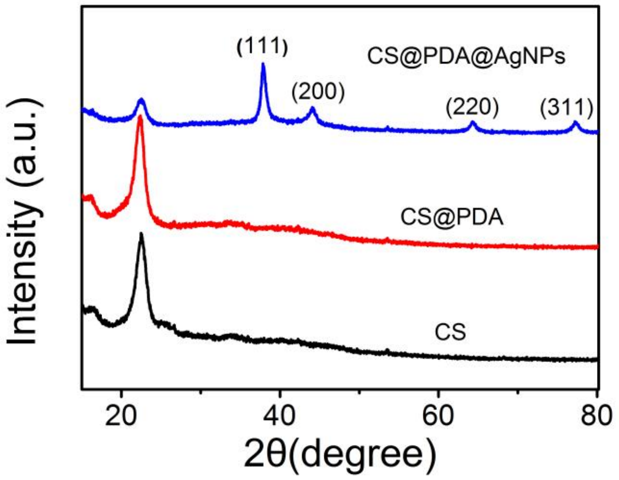

Figure S2. XRD of the original cotton swabs, CS@PDA swabs and CS@PDA@AgNPs swabs**.**

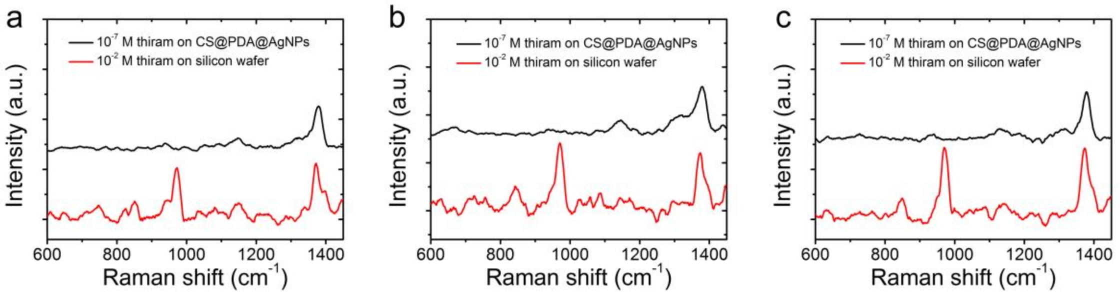

Figure S3. SERS spectra (black) of thiram on the CS@PDA@AgNPs swabs collected from (**a**) pears, (**b**) grapes and (**c**) peaches surface, and the Raman spectra (red) of thiram on silicon wafer substrate.
